# Cervicofacial flap for reconstruction of squamous cell carcinoma on the right cheek: A case report

**DOI:** 10.1002/ccr3.3379

**Published:** 2020-09-30

**Authors:** Shogo Ebisudani

**Affiliations:** ^1^ Department of Plastic and Reconstructive Surgery Kawasaki Medical School Kurashiki Japan

**Keywords:** cervicofacial flap, reconstruction of the cheek, squamous cell carcinoma

## Abstract

Reconstruction of the cheek to the lower eyelid can be performed with the use of a cervicofacial flap in patients that may not otherwise be able to sustain a long surgical procedure. In addition, the cervicofacial flap is superior in terms of color and texture match.

## Graphical abstract

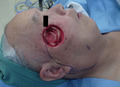

## 1 INTRODUCTION

There is a plethora of research on reconstruction methods for facial skin defects. A local skin flap is considered as a first‐choice treatment in many cases, from perspectives of color and texture match for the face. As reported by Juri in 1979, a cervicofacial flap is a highly useful option compared to other skin reconstruction methods because it allows the simultaneous use of the skin from the neck and the cheek.^1^, ^2^ In 1985, Jackson reported the use of a similar skin flap as a lateral cheek rotation flap.^3^ We experienced a case of a patient with squamous cell carcinoma (SCC) of the right cheek reconstructed with a cervicofacial flap.

## 2 CASE REPORT

**Figure 1:**
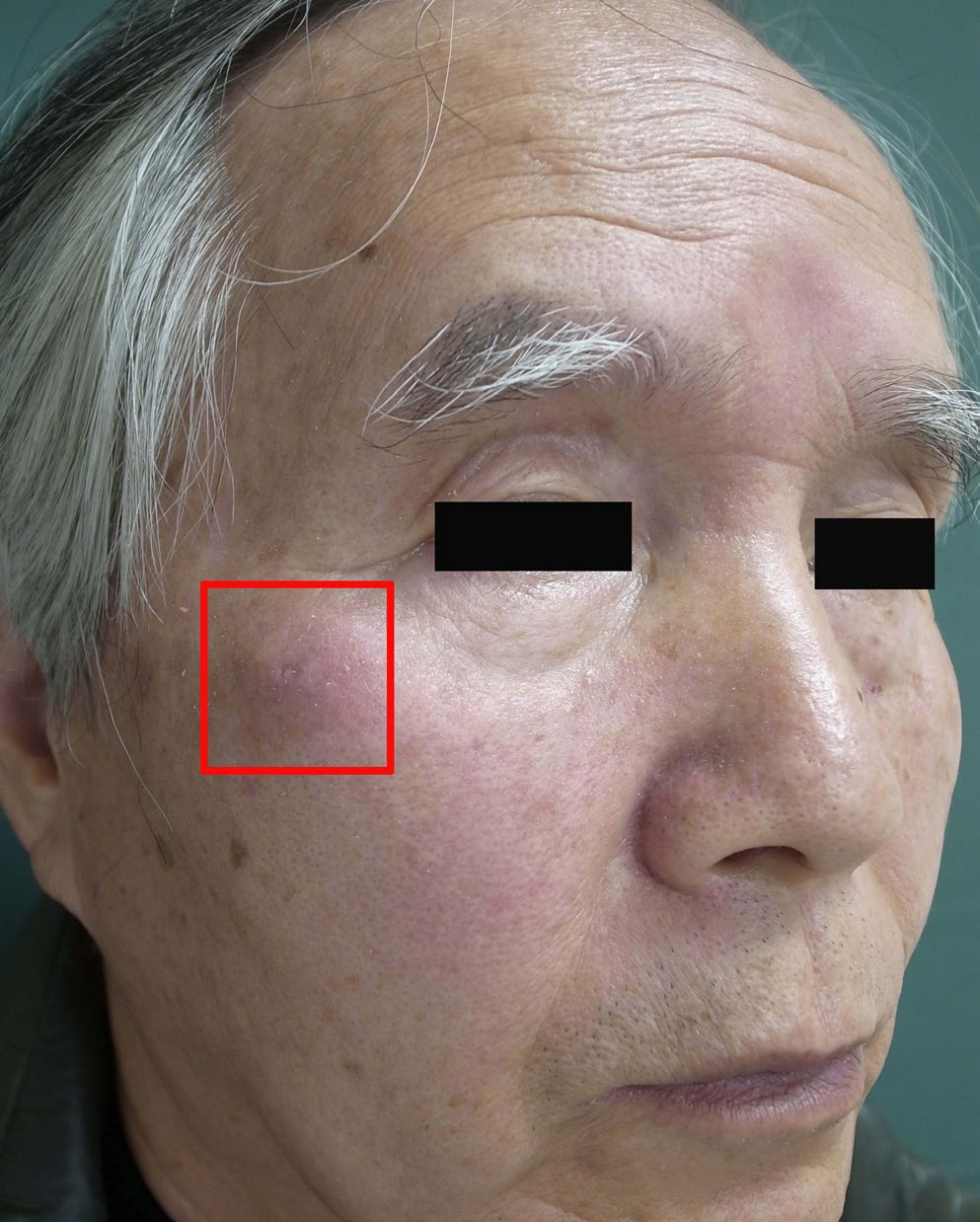
Clinical photograph on first visit. Subcutaneous tumor was observed in the framed area

The patient was a 69‐year‐old man. Two years prior, he developed an ulcer of the right cheek and received treatment with liquid nitrogen at a local clinic. The patient presented to our hospital, as he recently developed a subcutaneous mass on the right cheek, which gradually grew in size (Figure 1). He had a history of diabetes. He was receiving oral hypoglycemic agents and had good glycemic control, and we continued to administer these drugs to him.

**Figure 2:**
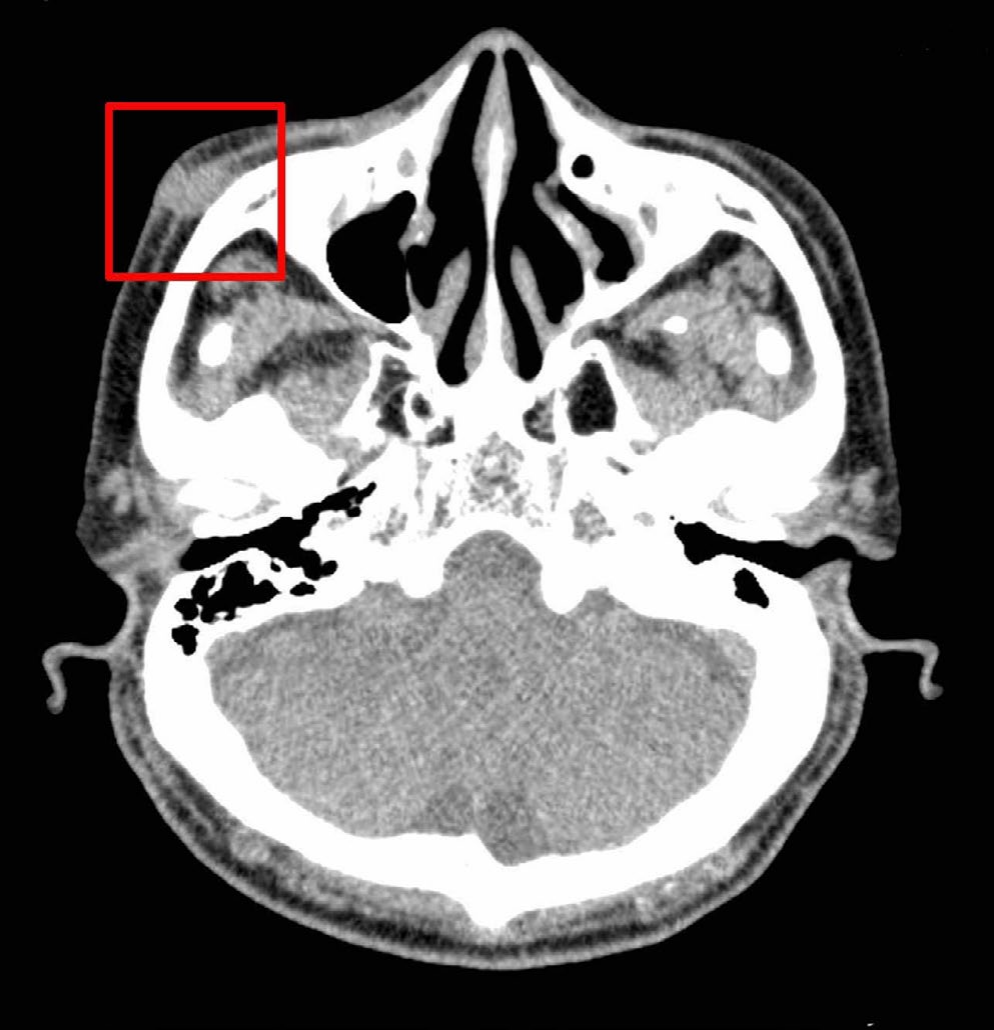
Plain CT image of the cheek. A mass in contact with the zygomatic bone can be observed in the framed area

**Figure 3:**
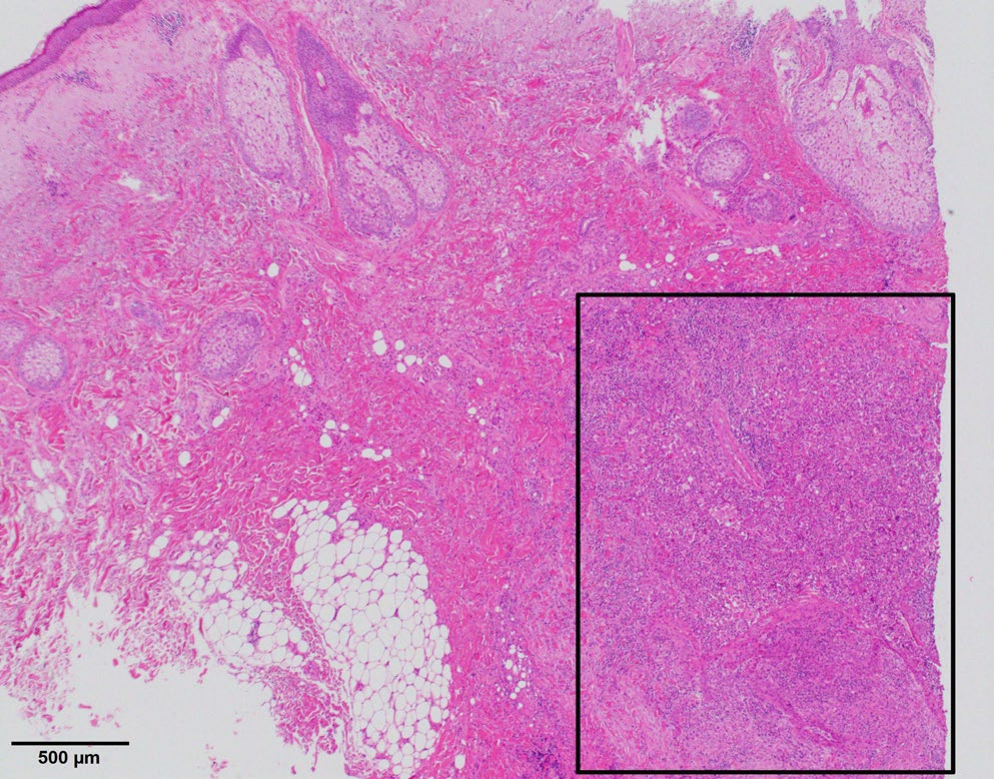
Microscopic examination (H&E stain). Confirmed well‐differentiated SCC was observed in the framed area

**Figure 4:**
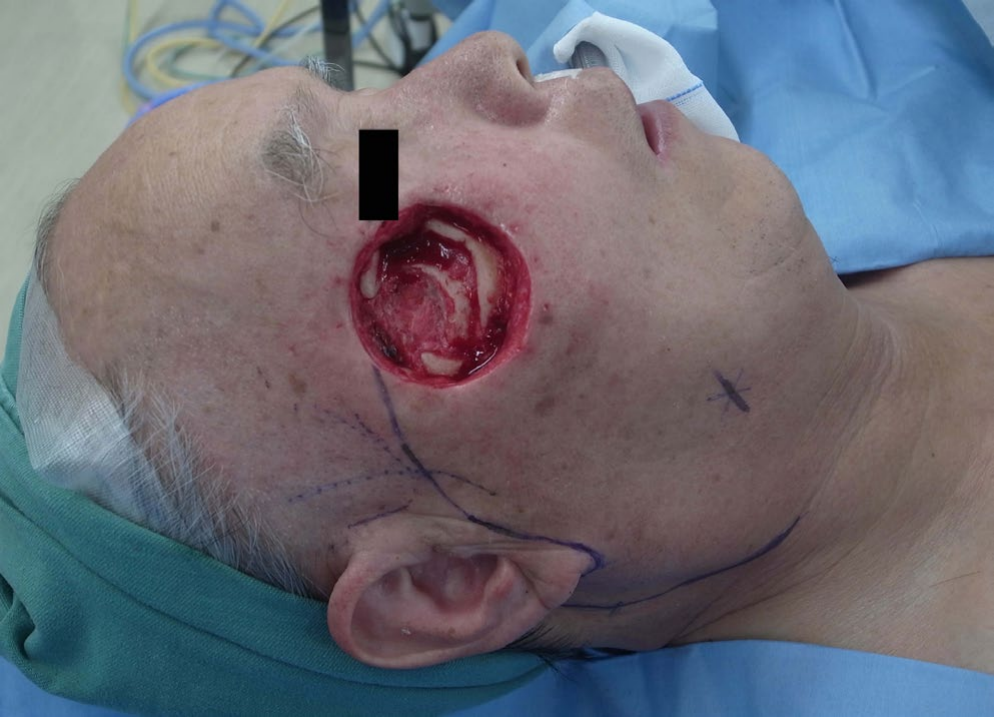
Image following tumor resection. Part of the zygomatic bone was removed. The design of the cervicofacial flap was drawn

**Figure 5:**
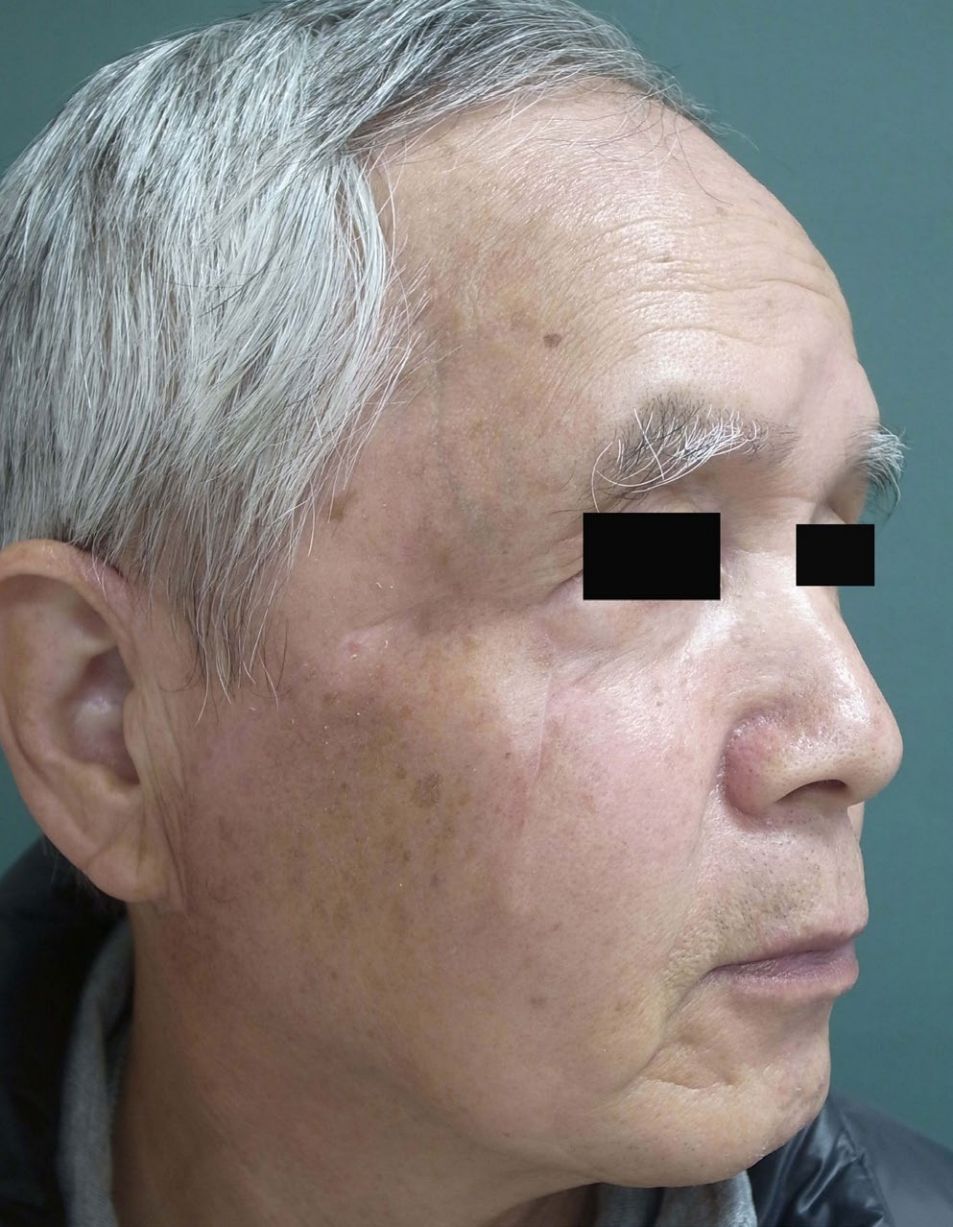
Fourteen months following surgery. Favorable color match and texture match were achieved

A mass of 1 cm in size with unclear margins was observed on plain computed tomography. The mass was in contact with the zygomatic bone (Figure 2). A biopsy was performed in March 2019, and the patient was pathologically diagnosed with SCC (Figure 3). As part of the mass was adhered to the zygomatic bone, we performed an extended radical excision with a 1.5‐cm margin from the excision scar in April 2019. The excision involved partial removal of the zygomatic bone. At that time, his glycemic control was satisfactory, and, therefore, we provided only oral hypoglycemic agents for diabetes. No residual SCC was observed in pathological examination. Therefore, this case was diagnosed as stage I with T1N0M0 in TNM staging. As complete excision of the tumor was confirmed, we performed a reconstruction of the tissue defect of the right cheek by using a cervicofacial flap. The incision line was drawn from the lateral side of the tissue defect through the area below the sideburn toward the earlobe (Figure 4). The skin flap was elevated above the superficial musculoaponeurotic system (SMAS) for the cheek and below the platysma for the neck. When elevating the skin flap, it is necessary to consider the marginal mandibular branch of the facial nerve, especially under the neck region, because the area under the platysma is exfoliated. The flap could completely cover the tissue defect. We placed a drain and closed the wound to prevent hematoma and seroma. Fourteen months have passed since the surgery, and no tumor relapse or ectropion of the lower eyelid has been observed. Additionally, very favorable outcomes have been achieved in terms of the color and texture match of the skin flap (Figure 5).

## 3 DISCUSSION

There is a plethora of research on the methods of reconstructing skin/soft tissue defects, including free skin graft, local flaps, and microvascular free flap tissue transfer. In the case of facial surgery, it is of great importance, from a perspective of postoperative aesthetics, to select a method that can achieve favorable outcomes in terms of color and texture match. From the 1980s, many studies have reported cases in which reconstruction was performed using a tissue expander.^4^, ^5^, ^6^ In contrast, local skin flaps may be the most optimal method when simplicity is the priority. We have recently used a cervicofacial flap in treating a large soft tissue defect accompanied by an exposed right zygomatic bone. The outcome of the surgery was favorable. Interestingly, a cervicofacial flap has the characteristics of a cheek and a cervical flap. It can cover a large defect wound on the face. As the skin flap tissue is adjacent to the tissue defect, this technique can achieve very good color and texture match. In addition, the suture line of the skin flap is aligned with the aesthetic units. Therefore, the postoperative scar will be almost imperceptible. A cervicofacial flap may be used to reconstruct a large defect wound in an older patient because the skin of many older adults is lax. Interestingly, microvascular free flap tissue transplantation is a lengthy surgical procedure. Therefore, many older patients and those with serious complications cannot undergo this operation. In contrast, a cervicofacial flap is a relatively simple operation. The method does not require special techniques, such as vascular anastomosis, and can shorten the surgery time. It has been reported that the method was performed under local anesthesia.^7^ In our case, as the zygomatic bone resection was relatively small, satisfactory results were obtained using cervicofacial flap. However, in cases where the zygomatic bone needs to be largely resected, it is considered necessary to perform microvascular free flap transfer, such as an osteocutaneous scapular flap.

Delay et al reported that dissecting the skin under the SMAS during cervicofacial flap elevation helps in addition to obtaining a thick skin flap and maintaining good blood flow in the skin flap.^8^ Meanwhile, Kaplan and Goldwyn reported that they dissected the neck skin above the platysma to avoid damaging the marginal mandibular branch of the facial nerve.^9^ We consider that it is still difficult to obtain a thicker flap for the cheek even if the skin is dissected under the SMAS. We rather consider that it could also increase the likelihood of damaging the facial nerves. In contrast, regarding the neck, it is possible to dissect the platysma while checking the marginal mandibular branch of the facial nerve. Hence, to achieve the appropriate blood flow and thickness for the skin flap, we dissected the skin under the platysma. As another complication, this surgery may cause blood flow disturbances in the skin around the auricle and the ear lobe.^10^, ^11^, ^12^ Lim et al^13^ reported that the incidence of complications in cervicofacial flap surgery was 7.1%. Additionally, desensitization of the earlobe may occur in cases where the great auricular nerve is damaged when the posterior ear is incised. To avoid such complications, it is necessary to consider when incising the area around the auricle. The benefit of cervicofacial flaps significantly outweighs such complications, and it is considered as a highly useful material for reconstruction of the area from the cheek to the lower eyelid.

## 4 CONCLUSION

We have recently experienced a case of a patient with SCC of the right cheek that required partial removal of the right zygomatic bone. We performed a reconstruction with a cervicofacial flap for this patient. Cervicofacial flaps helped us to shorten the surgery time. They can be also used for older patients and those with complex comorbidities. In addition, cervicofacial flaps are superior in terms of color and texture match. Hence, it was considered that they are a highly useful material for reconstruction of the area ranging from the cheek to the lower eyelid.

## CONFLICT OF INTEREST

The authors declare that they have no conflict of interest.

## AUTHOR CONTRIBUTIONS

SE: wrote the article and has accountability for all aspects of the work.

## ETHICAL APPROVAL

The patient gave written consent to report his case and the imaging.
